# Social participation and depressive symptoms of carer-employees of older adults in Canada: a cross-sectional analysis of the Canadian Longitudinal Study on Aging

**DOI:** 10.17269/s41997-021-00524-5

**Published:** 2021-06-10

**Authors:** Li Wang, Chris Ji, Peter Kitchen, Allison Williams

**Affiliations:** 1 Offord Centre for Child Study & Department of Psychiatry and Behavioural Neurosciences, McMaster University, Hamilton, Ontario Canada; 2 Department of Mathematics, University of Waterloo, Waterloo, Ontario Canada; 3 School of Earth, Environment and Society, McMaster University, 1280 Main Street West, Hamilton, Ontario L8S 4K1 Canada

**Keywords:** Depressive symptoms, Carer-employees, Social participation, CLSA, Symptômes dépressifs, soignants-employés, participation sociale, ELCV

## Abstract

**Objectives:**

This study used two waves of data from the Canadian Longitudinal Study on Aging (CLSA) to investigate the association between social participation and depressive symptoms in carer-employees (CEs) and non-carer-employees (NCEs).

**Methods:**

Adopting Pearlin et al.’s stress model, multivariate linear regression was used to examine the relationships among carer role, social participation, and depressive symptoms in Canadian employees using the first two waves of CLSA data, while controlling for possible confounders.

**Results:**

Higher levels of social participation were found to be associated with lower depressive symptoms in both waves. Social participation was found to moderate depressive symptoms for CEs when compared with NCEs in Wave 2 but not in Wave 1.

**Conclusion:**

The present study highlights the importance of social participation in reducing CEs’ depressive symptoms. The findings provide support for innovative policy and intervention efforts to encourage and enhance social participation at work via carer-friendly workplace policies for CEs across Canada.

## Résumé

**Objectifs:**

Cette étude a utilisé deux vagues de données de l’Étude longitudinale canadienne sur le vieillissement (ÉLCV) pour étudier l’association entre la participation sociale et les symptômes dépressifs chez les employés soignants (ES) et les employés non soignants (ENS).

**Méthodes:**

Adoptant le concept du modèle de stress de Pearlin et al., une régression linéaire multivariée a été utilisée pour examiner les relations entre le rôle de l’aidant, la participation sociale et les symptômes dépressifs chez les employés canadiens en utilisant les deux premières vagues de données de l’ÉLCV, tout en contrôlant leurs facteurs de confusion.

**Résultats:**

Des niveaux plus élevés de participation sociale se sont avérés être associés à des symptômes dépressifs plus faibles de façon constante dans les deux vagues. Au fil du temps, la participation s’est avérée modérer les symptômes dépressifs pour les ES par rapport aux ENS dans la vague 2 mais pas dans la vague 1.

**Conclusion:**

La présente étude souligne l’importance de la participation sociale en ce qui concerne l’atténuation des symptômes dépressifs des ES. Les résultats appuient les efforts politiques et d’intervention visant à encourager et à améliorer la participation sociale au travail des ES partout au Canada.

## Introduction

Carer-employees (CEs) simultaneously balance paid work and unpaid caring demands. A recent survey showed that approximately 35% of the Canadian workforce had provided care to elderly family members or friends during the preceding year (Sinha, 2013). CEs significantly contribute to the Canadian health care system by providing a substantial amount of unpaid care. Their workplace participation provides both financial and social support to their unpaid caring role. However, work stress can compound with stress due to caring, leading to negative outcomes, particularly for women (Williams et al., 2017). There is a growing tendency for carers to report feelings of distress, anger or depression, and of being unable to continue to provide care; 15.6% of carers felt this way in 2009–2010, and 33.3% in 2013–2014 (Health Quality Ontario, 2017).

Depressive symptoms have shown a strong relationship to the carer experience (Day, 2017), although some studies downplay this correlation (Robison et al., 2009). With that said, the consensus is that there are significantly higher rates of depression and stress in carers than in non-carers, and this is magnified in dementia carers (Wang et al., 2011). The limitation of many such studies is that the non-carers in the general population were commonly used as the control group. As O’Reilly et al. (2008) interpret, this may be the ‘healthy worker effect’ phenomenon, with those with physical limitations and/or poor health self-selecting out of the caring role and/or labour force. Sibalija et al. (2020) attempted to match the non-carers and informal carers based on age, gender and education; however, the ‘healthy worker effect’ was still not ruled out while examining the role of caring on depression. The pre-existing health disparities (beyond those accounted for by age and sex) between carers and non-carers might give rise to misleading conclusions about the association between employment and carers’ depression. Thus, there is a critical need for researchers to better understand the mental health of CEs in Canada. In this study, we examine the caring role on depression within a sample of Canadians who were active in the labour market.

Previous studies have shown that social participation in community-based activities (i.e., cultural/educational activities, recreational sports) is an important component of health. Due to caring responsibilities and paid work, CEs often must miss opportunities for social participation, giving up hobbies and recreational activities. This can lead to enhanced stress, depression, and feelings of isolation, as well as limited time alone with family (Wang et al., 2018). Consequently, it is important to assess whether carers’ social participation exhibits any modifying or protective effect on their depressive symptoms. While it can be rewarding to care for a loved one, the negative health impacts may snowball over time. The two waves of the Canadian Longitudinal Study on Aging (CLSA) provide us an opportunity to examine the association between social participation and CE depression. Through a parallel analysis (cross-sectional) of two waves of CLSA data, this study provides a better understanding of how social participation influences Canadian CEs’ depressive symptoms.

### Theoretical framework

We adopt Pearlin et al.’s (1990) stress process model of caring for CEs, while also understanding the moderate effect of social participation on carers’ depressive symptoms. The model understands carer stress as a process comprising a number of interrelated conditions, including the socio-economic characteristics and resources of carers, as well as a range of stressors to which they are exposed. Primary stressors are defined in the model as hardships and problems stemming directly from caring. Secondary stressors are categorized as either the strains experienced in roles/activities external to caring or intrapsychic strains which involve the diminishment of self-concept. Certainly, the work CEs do in balancing both informal family carer and employee roles operates as a secondary stress in this model. Pearlin et al. (1990) emphasize the negative impact of role conflict as a secondary stressor, and the positive impact of social support as a protective factor which reduces the negative outcomes of stressors. As noted by Sibalija et al. (2020), social participation can be a source of social support, the latter which is a modifiable moderator that intervenes at multiple points along the stress process, affecting the strength of the relation between stressors and negative outcomes (Pearlin et al., 1990).

Population-level evidence examining the association between social participation and depressive symptoms for CEs is extremely limited in Canada and elsewhere. Better understanding these relationships using the large data set available via the CLSA has the potential to inform the design and implementation of interventions that enhance protective factors in both the workplace and community.

### Objectives

The primary objective of this study is to understand the linkages among caring, social participation and depressive symptoms among Canadian employees using parallel analysis of two waves of the Canadian Longitudinal Study on Aging (CLSA), controlling for a range of possible confounders. The study addresses three questions: (1) Are higher levels of social participation associated with lower depressive symptoms for both carer and non-carer employees? (2) Is the association between carer status and depressive symptoms moderated by social participation?; and, if so, (3) Does the moderation effect of social participation on depressive symptoms change from Wave 1 to Wave 2?

## Method

### Data source

We obtained data from two waves of the CLSA, a nationally representative sample of 21,241 participants (CLSA, 2011), targeting Canadians between the ages of 45 and 85 across the country. Data for Wave 1 (*n* = 21,241) were collected between 2011 and 2015, and for Wave 2 (*n* = 17,052) between 2015 and 2018. The target population in this study were those active in the Canadian labour force. Thus, we used the entirety of the CLSA to select participants who were either currently working or considered themselves either partly retired or not retired based on the questions “What is your current working status?” and “At this time, do you consider yourself to be completely retired, partly retired, or not retired?” From the selected participants, a sample size of Canadian employees who participated in Wave 1 were tracked in Wave 2 (*n* = 6914). Next, the subsample of employees who were simultaneously providing informal care (i.e., the CEs) was determined. The CEs were participants who reported that they had aided another person within the past 12 months. However, research suggests that there are significant differences between carers of children (usually performed by young parents) and carers of older adults (usually performed by spouses, sons or daughters) with respect to carer burden, mental health, and physical function (de Oliveira et al., 2015). Due to our interest in carers of older adults, we chose to focus on older CEs. Given that the CLSA data set lacks information on care recipients’ age, we identified the carers of older adults based on the question specific to the relationship with the care recipients. Participants were asked “What is the relationship between you and [person you provided most caregiving assistance to]”. Those who were caring for someone in the same generation or above (spouse, common-law partner, sibling, sibling-in-law, parent, parent-in-law or grandparent) were considered the targeted carer participants in this study. We excluded 218 carers of children (son, daughter, grandson and granddaughter) from Wave 1 data, and 288 from Wave 2 data. As a result, we used a sample size of 6626 from Wave 1, who were tracked in Wave 2.

### Measures

#### Depressive symptoms

In the CLSA, depressive symptoms were measured using the Center for Epidemiological Studies Short Depression Scale (CESD) (Radloff, 1977). This is a self-report measure of depressive symptoms found by summing the answers to 10 different questions on how frequently in the past 7 days they experienced the symptoms of depression, such as being easily bothered, trouble concentrating, feeling depressed, and feeling fearful and tearful. Each question was rated on a 4-point scale from 0 to 3, where the range of the scale was 0–30. Higher scores indicate higher levels of depressive symptoms. Cronbach’s alpha for the 10-item scale is 0.719.

#### Social participation

Social participation in the CLSA is measured by the frequency of participating in seven different community-related activities in the last 12 months, such as participating in religious activities, neighbourhood activities, or educational/cultural activities. Frequencies were scored on a 5-point scale from 0 (never) to 4 (at least once a day), with a higher number indicating greater social participation. We summed these to create a total social participation score with a range of 0–28. Cronbach’s alpha for the 7 items in the study sample is 0.541.

#### Control variables

Research suggests that experiencing higher stress or depression is associated with where the care recipient lives, whether in an institution, within the family home or in their own home (Duxbury et al., 2011). Thus, we created a dummy variable with 1 indicating that care recipients were living in the same house as the CEs. In addition to age, sex and educational attainment, the study also controlled for immigrant status, work status (operationalized below as number of hours worked/week) and household income, which are potential confounding factors in being able to access forms of social participation (Arai et al., 2014; Luchsinger et al., 2015; Kim et al., 2011). Educational attainment was grouped into three categories: (a) secondary and below, (b) some sort of post-secondary degree, and (c) bachelor’s and above. Work status was categorized based on the hours worked in a week: 30 h and above, between 20 and 30 h, and less than 20 h. Household income includes five categories ranging from less than $20,000 to more than $150,000. In addition, we controlled for perceived health given that poor physical health is associated with a higher risk of developing depressive symptoms (Arai et al., 2014; Luchsinger et al., 2015; Piercy et al., 2013). Perceived health was dichotomized into favourable (excellent and very good) and unfavourable (poor, fair and good) health based on a single question on self-rated general health. Health care access was controlled for given that access to comprehensive, quality health care is associated with better mental and physical health (Caldwell, 2008). We created four dichotomous variables, with 1 indicating having at least one visit to a (1) family doctor, (2) medical specialist, (3) dentist and/or (4) psychologist/social worker in the past 12 months, respectively.

#### Analysis

Overall, 19.7% of study variables included missing values in Wave 1 data and 12.5% in Wave 2 data. To reduce the risk of bias from missing data, multiple imputation by chained equations (MICE) was performed to impute values for the missing data. Primary analysis was based on 10 multiple imputed data sets, and results were combined using Rubin’s combination rules (Rubin, 2004). A comparison of the estimates from imputed and complete case analysis produced consistent results.

All analyses used sampling weights to generate the estimation representative of the target population of Canadian employees. We also used a paired *t* test to examine the differences in depressive symptoms and social participation across waves, as well as the *t* test for the difference between CEs and non-carer-employees (non-CEs) within each wave. Multivariate linear regressions were run in parallel using Wave 1 and Wave 2 separately. Inspection of the distribution of continuous variables reflecting the total amount of depression symptoms participants reported was slightly non-normal, with skewness and kurtosis as follows: Wave 1, skew = 1.50, kurtosis, 5.56; Wave 2, skew = 1.26, kurtosis = 4.52. As transforming variables to improve normality makes interpretation difficult, we decided to use the original scale but estimated the robust standard error using the Huber-White sandwich estimators that take into account issues concerning heterogeneity and lack of normality. Models 1 and 2 estimate the association between social participation and depressive symptoms for CEs and non-CEs, respectively. Model 3 estimates the extent to which social participation moderates the association between carer status and depressive symptoms by testing the interaction term of social participation and carer status. All models include a range of control variables under consideration.

Our sample includes the participants who cared for ‘other’ people (other relative, friend, neighbour, or other). We conducted a sensitivity analysis in order to examine whether our results would be influenced by including those participants. STATA 14.0 was used for all data analyses (StataCorp, College Station, TX, USA 2015).

## Results

### Descriptive findings

Our sample includes 6626 CLSA participants (CEs and non-CEs) who were in both Wave 1 and Wave 2 data. Table 1 presents the weighted descriptive characteristics of the variables used in the analysis by caregiver status in each wave, along with their corresponding difference test. Of the 6626 participants, 2268 were CEs in Wave 1 and 2616 were CEs in Wave 2. The average depressive symptom scores in the two waves were similar, with the value of 5.04. Specifically, on average, approximately 10% of participants (Wave 1: 10.3%; Wave 2: 10.6%) experienced the symptoms of depression occasionally, and around 4% (Wave 1: 3.8%; Wave 2: 3.9%) all the time (Table 2). However, the difference between CEs and non-CEs is statistically significant in Wave 1, but not in Wave 2. The average social participation scores in full Wave 1 were higher than those in full Wave 2 (10.82 vs. 9.15). In contrast, CEs and non-CEs had the same level of social participation in Wave 1 (10.81 vs. 10.85), but CEs showed significantly higher social participation scores in Wave 2 (8.95 vs. 9.44). In both waves, respondents who were CEs were significantly more likely than non-CE respondents to be younger and female and have secondary education or above, and to have engaged in psychologist/social worker visits. Additionally, comparing full Wave 1 with full Wave 2, the overall proportion of ‘working more than 30 h’ (82.3% vs. 73.2%) and ‘perceived favourable health’ (64.0% vs. 60.8%) significantly decreased; however, the proportion of household income above $150,000 significantly increased from 23.0% in Wave 1 to 26.3% in Wave 2. There were also a number of other variables that increased significantly from full Wave 1 to full Wave 2, including the overall proportion of care recipients living in the same house as the carer (8.5% vs. 11.1%) and the overall incidences of visits to medical specialists (39.1% vs. 49.5%) and dentists (84.0% vs. 87.1%).

**Table 1:** Descriptive statistics of sample characteristics

	Wave 1 data (*N* = 6626)				Wave 2 data (*N* = 6626)				Wave 1 vs. Wave 2
	Full	Non-CEs (*N* = 4358)	CEs (*N* = 2268)	Non-CEs vs. CEs	Full	Non-CEs (*N* = 4010)	CEs (*N* = 2616)	Non-CEs vs. CEs	
Variables	%	%	%	*t* test	%	%	%	*t* test	Paired *t* test
Depression symptom score (M, SE)	5.04 (0.05)	4.95 (0.05)	5.21 (0.06)	2.41 (*p* = 0.02)	5.04 (0.06)	5.02 (0.06)	5.06 (0.06)		
Social participation score (M, SE)	10.82 (0.05)	10.81 (0.05)	10.85 (0.05)		9.15 (0.05)	8.95 (0.05)	9.44 (0.04)	5.27 (*p* < 0.001)	
Carer status	36.3				41.7				
Care recipient in same house as carer	8.5	0	22.2		11.1	0	25.1		4.87 (*p* < 0.001)
Age (M, SE)	53.87 (0.08)	54.24 (0.08)	53.21 (0.07)	− 6.37 (*p* < 0.001)	58.07 (0.08)	58.74 (0.08)	57.14 (0.07)	− 10.24 (*p* < 0.001)	
Sex (female)	46.6	44.2	50.7	5.14 (*p* < 0.001)	46.6	44.2	50.7	5.27 (*p* < 0.001)	
Education									
Secondary and below	33.7	35.2	31.1	− 3.48 (*p* = 0.001)	33.8	35.8	30.9	− 4.08 (*p* < 0.001)	
Some sort of post-secondary	25	23.5	27.5	3.69 (*p* < 0.001)	24.9	24.2	26	1.69 (*p* = 0.09)	
Bachelor’s or above	41.3	41.3	41.4		41.3	40	43.1	2.43 (*p* = 0.01)	
Household income									
Less than $20,000	2.1	2.4	1.7		1.9	2.3	1.2	− 3.11 (*p* = 0.002)	
$20,000–$49,999	13.4	14.8	11.1	− 4.07 (*p* < 0.001)	14.4	15.7	12.5	− 3.62 (*p* < 0.001)	
$50,000–$99,999	35.5	35.1	36.2		31.5	32.0	30.9		− 4.49 (*p* < 0.001)
$100,000–$149,999	25.9	24.4	28.5	3.57 (*p* < 0.001)	25.9	25.4	26.6		
$150,000 or more	23.0	23.3	22.5		26.3	24.5	28.7	3.74 (*p* < 0.001)	4.21 (*p* < 0.001)
Work status									
More than 30 h a week	82.3	82	82.9		73.2	73.1	73.3		9.66 (*p* < 0.001)
Between 20 and 30 h a week	10	10.3	9.5		11.7	10.4	13.5	3.35 (*p* = 0.001)	
Less than 20 h a week	7.7	7.7	7.6		15.2	16.6	13.3	− 3.67 (*p* < 0.001)	10.16 (*p* < 0.001)
Perceived health (favourable)	64	63.8	64.4		60.8	60.1	61.9		
Access to health care									
Family doctor access	84.9	84.7	85.4		86.4	86.1	86.7		
Medical specialist access	39.1	39	39.2		49.5	50.5	48.1	− 1.87 (*p* = 0.06)	12.01 (*p* < 0.001)
Dentist access	84	83.6	84.8		87.1	86.7	87.8		7.93 (*p* < 0.001)
Psychologist/social worker access	7.2	6.6	8.2	2.49 (*p* = 0.01)	8.1	7.4	9	2.35 (*p* = 0.02)

**Table 2:** The frequency of the CES-D 10 scale for Wave 1 and Wave 2

	Rarely or never		Some of the time		Occasionally		All of the time	
	Wave 1 (%)	Wave 2 (%)	Wave 1 (%)	Wave 2 (%)	Wave 1 (%)	Wave 2 (%)	Wave 1 (%)	Wave 2 (%)
Easily bothered	65.4	68.7	19.1	17.3	12.8	11.7	2.7	2.3
Trouble concentrating	61.7	61.3	20.2	20.2	14.2	14.6	3.9	3.9
Feel depressed	80.6	79.5	12.2	11.8	5.7	7	1.5	1.6
Feel everything is an effort	69.2	71	17.9	16.8	9.1	8.8	3.7	3.5
Feel hopeless about the future	61.6	61.5	22.9	21.8	10.5	11.6	5	5.1
Feel fearful or tearful	76.9	76.6	14.9	13.9	6.8	7.4	1.4	2.1
Sleep is restless	38	37.7	28	27.7	20.4	20.5	13.7	14.1
Feel not happy	64.8	65.7	25.3	24	8.1	8.4	1.9	1.9
Feel lonely	81.4	81.5	10.6	9.8	6.1	6.9	1.9	1.8
Feel could not ‘get going’	72.2	70.9	16.6	17.2	9	9	2.2	3
Average	67.2	67.4	18.8	18.1	10.3	10.6	3.8	3.9

### Multivariate linear regression

Table 3 presents the multivariate linear regression models on depressive symptoms using the Wave 1 and Wave 2 data, respectively. The unstandardized beta coefficients are reported when presenting the findings of the regression. The first two models estimate the association between social participation and depressive symptoms, separately for non-CEs and CEs at each wave, controlling for all confounders. The results show that social participation was significantly associated with decreased depressive symptoms for both non-CEs (*B* = − 0.11; *p* < 0.001) and CEs (*B* = − 0.14; *p* < 0.001) in Wave 1, and again in Wave 2 (*B* = − 0.09; *p* < 0.001 for non-CEs vs. *B* = − 0.15; *p* < 0.001 for CEs). Using the full sample, the third model included the main effects of carer status and social participation, as well as their interaction for both Wave 1 and Wave 2 data. CEs were more likely to report higher depressive symptoms in Wave 1 (*B* = 0.62; *p* < 0.05) and in Wave 2 (*B* = 0.63; *p* < 0.05) than the non-CEs. Also, in the model using the full sample, more frequent social participation was consistently associated with decreased depressive symptom scores in both Wave 1 (*B* = − 0.11; *p* < 0.001) and Wave 2 models (*B* = − 0.09; *p* < 0.001). The interaction between social participation and carer status was negatively significant in Wave 2. Specifically, it shows that social participation was a significant modifiable factor (*B* = − 0.06; *p* < 0.05) to intervene between the carer role and depressive symptoms; that is, CE depression scores would be decreased by 0.06 with one unit increase in the social participation score. However, the moderation effect of social participation was not significant in Wave 1. At both waves, the results of the control variables show that being female, having a lower household income, and having unfavourable general health are positively related to depressive symptoms for CEs, non-CEs and the full sample. Using the sample of CEs, those who had their care recipient living in the same house were negatively associated with depressive symptoms (*B* = − 0.46, *p* < 0.05) when compared with those who did not in Wave 1; the association was not as significant in Wave 2.

**Table 3:** Multivariate linear regression on depressive symptom scores

	Wave 1 data (*N* = 6626)			Wave 2 data (*N* = 6626)		
	Non-CEs (*N* = 4358)	CEs (*N* = 2268)	Full (*N* = 6626)	Non-CEs (*N* = 4010)	CEs (*N* = 2616)	Full (*N* = 6626)
	Coef. (SE)	Coef. (SE)	Coef. (SE)	Coef. (SE)	Coef. (SE)	Coef. (SE)
Social participation	− 0.11 (0.02)***	− 0.14 (0.03)***	− 0.11 (0.02)***	− 0.09 (0.02)***	− 0.15 (0.02)***	− 0.09 (0.02)***
Carer-employees			0.62 (0.3)**			0.63 (0.28)**
Social participation × carer-employees			− 0.04 (0.03)			− 0.06 (0.03)**
Carer recipient in same house		− 0.46 (0.22)**	− 0.37 (0.21)*		0.16 (0.18)	0.18 (0.18)
Age	− 0.05 (0.01)***	− 0.02 (0.02)	− 0.04 (0.01)***	− 0.04 (0.01)***	− 0.03 (0.02)**	− 0.03 (0.01)***
Sex (female)	0.57 (0.13)***	0.69 (0.19)***	0.62 (0.11)***	0.76 (0.14)***	0.73 (0.16)***	0.74 (0.1)***
Immigrant	− 0.21 (0.17)	0.12 (0.28)	− 0.11 (0.15)	0.35 (0.18)**	0.01 (0.25)	0.24 (0.14)
Household income (ref: $150,000 or more)						
$100,000–$149,999	0.19 (0.19)	0.03 (0.27)	0.13 (0.15)	0.1 (0.19)	0.08 (0.22)	0.09 (0.14)
$50,000–$99,999	0.49 (0.18)***	0.67 (0.27)**	0.56 (0.15)***	0.67 (0.19)***	0.55 (0.22)**	0.61 (0.15)***
$20,000–$49,999	0.99 (0.23)***	1.31 (0.39)**	1.09 (0.2)***	1.28 (0.25)***	1.38 (0.3)***	1.3 (0.2)***
Less than $20,000	2.84 (0.46)***	1.7 (0.96)*	2.54 (0.44)***	3.76 (0.51)***	1.47 (0.74)**	3.09 (0.43)***
Education: (ref: ≤ secondary)						
Some sort of post-secondary	0.22 (0.17)	0.15 (0.24)	0.19 (0.14)	0.47 (0.17)**	− 0.23 (0.21)	0.18 (0.13)
Bachelor’s or above	0.28 (0.16)	0.35 (0.23)	0.3 (0.13)**	0.1 (0.17)	− 0.08 (0.2)	0.03 (0.13)
Work status: (ref: more than 30 h)						
20–30 h worked	0.53 (0.24)**	0.25 (0.35)	0.44 (0.19)**	0.36 (0.23)	0.01 (0.25)	0.18 (0.17)
<20 h worked	0.51 (0.3)	0.33 (0.4)	0.46 (0.24)	0.13 (0.21)	0.06 (0.28)	0.1 (0.17)
General health (favourable)	− 1.78 (0.13)***	− 1.89 (0.19)***	− 1.82 (0.11)***	− 2.22 (0.14)***	− 2.13 (0.17)***	− 2.18 (0.11)***
Family doctor access	0.29 (0.18)	0.53 (0.26)**	0.38 (0.15)**	0.22 (0.19)	0.62 (0.23)***	0.38 (0.15)***
Medical specialist access	0.52 (0.13)***	0.5 (0.19)**	0.5 (0.11)***	0.1 (0.13)	0.71 (0.16)***	0.36 (0.1)***
Dentist access	0.15 (0.17)	− 0.29 (0.26)	− 0.01 (0.14)	0.23 (0.25)	− 0.39 (0.26)	− 0.04 (0.18)
Psychologist or social worker access	1.98 (0.25)***	1.53 (0.34)***	1.78 (0.2)***	2.08 (0.25)***	2.68 (0.27)***	2.37 (0.18)***
*R*^2^ (%)	12.8	14.0	13.5	12.9	13.8	14.2

Significant level: ***p < 0.01, **p < 0.05, *p < 0.1

The sensitivity analysis was conducted by dropping the participants who cared for ‘other’ people (other relative, friend, neighbour, or other) and shows our results are robust. The results are reported in Table 4.

**Table 4:** Sensitivity analysis: multivariate linear regression on depressive symptom scores

	Wave 1 data (*N* = 5812)			Wave 2 data (*N* = 5584)		
	Non-CEs (*N* = 3544)	CEs (*N* = 2268)	Full	Non-CEs (*N* = 2968)	CEs (*N* = 2616)	Full
	Coef. (std. err.)	Coef. (std. err.)	Coef. (std. err.)	Coef. (std. err.)	Coef. (std. err.)	Coef. (std. err.)
Social participation	− 0.15 (0.02)***	− 0.14 (0.03)***	− 0.14 (0.02)***	− 0.09 (0.02)***	− 0.15 (0.02)***	− 0.09 (0.02)***
Carer-employees			0.47 (0.32)			0.68 (0.29)**
Social participation × carer-employees			− 0.01 (0.03)			− 0.06 (0.03)**
Carer recipient in same house		− 0.46 (0.22)**	− 0.39 (0.21)*		0.16 (0.18)	0.2 (0.18)
Age	− 0.06 (0.01)***	− 0.02 (0.02)	− 0.04 (0.01)***	− 0.05 (0.01)***	− 0.03 (0.02)**	− 0.04 (0.01)***
Sex (female)	0.49 (0.14)***	0.69 (0.19)***	0.58 (0.12)***	0.84 (0.16)***	0.73 (0.16)***	0.77 (0.11)***
Immigrant	− 0.21 (0.19)	0.12 (0.28)	− 0.1 (0.16)	0.42 (0.2)**	0.01 (0.25)	0.26 (0.16)
Household income (ref: $150,000 or more)						
$100,000–$149,999	− 1.37 (0.55)**	− 0.4 (1.04)	− 1.03 (0.52)*	− 2.71 (0.69)***	− 0.09 (0.75)	− 1.59 (0.53)***
$50,000–$99,999	− 2.08 (0.54)***	− 1.03 (0.95)	− 1.7 (0.51)***	− 3.49 (0.67)***	− 0.91 (0.73)	− 2.4 (0.51)***
$20,000–$49,999	− 2.38 (0.55)***	− 1.67 (0.97)*	− 2.15 (0.52)***	− 3.72 (0.69)***	− 1.38 (0.74)*	− 2.74 (0.52)***
Less than $20,000	− 2.26 (0.56)***	− 1.7 (0.96)*	− 2.08 (0.52)***	− 4.23 (0.7)***	− 1.47 (0.74)**	− 3.03 (0.53)***
Education: (ref: ≤ secondary)						
Some sort of post-secondary	0.08 (0.19)	0.15 (0.24)	0.12 (0.15)	0.38 (0.2)*	− 0.23 (0.21)	0.08 (0.14)
Bachelor’s or above	0.26 (0.17)	0.35 (0.23)	0.3 (0.14)**	0.16 (0.19)	− 0.08 (0.2)	0.05 (0.14)
Work status: (ref: more than 30 h)						
20–30 h worked	0.52 (0.27)	0.25 (0.35)	0.41 (0.21)*	0.28 (0.28)	0.01 (0.25)	0.12 (0.19)
<20 h worked	0.77 (0.33)***	0.33 (0.4)	0.59 (0.24)	0.14 (0.25)	0.06 (0.28)	0.12 (0.19)
General health (favourable)	− 1.67 (0.15)***	− 1.89 (0.19)***	− 1.77 (0.12)***	− 2.11 (0.16)***	− 2.13 (0.17)***	− 2.11 (0.11)***
Family doctor access	0.38 (0.2)*	0.53 (0.26)**	0.45 (0.16)***	0.3 (0.22)	0.62 (0.23)***	0.45 (0.16)***
Medical specialist access	0.49 (0.14)***	0.5 (0.19)**	0.48 (0.12)***	− 0.01 (0.15)	0.71 (0.16)***	0.34 (0.11)***
Dentist access	0.35 (0.19)*	− 0.29 (0.26)	0.09 (0.15)	0.22 (0.31)	− 0.39 (0.26)	− 0.08 (0.19)
Psychologist or social worker access	2.25 (0.27)***	1.53 (0.34)***	1.91 (0.21)***	1.89 (0.29)***	2.68 (0.27)***	2.35 (0.2)***
*R*^2^	11.40	9.15	10.08	12.7	15.75	13.73

Participants who cared for ‘other’ people (other relative, friend, neighbour, or other) were dropped from the sample

Significant level: ***p < 0.01, **p < 0.05, *p < 0.1

## Discussion

Framed within Pearlin et al.’s stress model (1990), this paper presents a study of individual-level experience of depressive symptoms for CEs, in relation to social participation, via the analysis of two waves of the CLSA data. It is the first study that follows CEs from 2010 to 2014, allowing us to examine the association between CEs and depressive symptoms and to better understand the moderation effect of social participation, via two cross-sectional analyses of CLSA data respectively. We found that the characteristics of the CLSA participants in the full sample significantly varied from Wave 1 to Wave 2, with respect to household income, work status, perceived general health, and health service use. The participants tended to work fewer hours but had higher household income, likely the result of their pensions starting at age 65 for some participants and their family members. We observed that the proportion of participants who reported their income from the Canada or Quebec pension plans increased by 5% (Wave 1: 22.3% vs. Wave 2: 27.4%). Furthermore, participants in Wave 2 had poorer physical health with increasing age, thus had a greater number of health care visits. This study has added value to the literature on CEs’ mental health (Wang et al., 2018; Robison et al., 2009).

The descriptive statistics show that there is no significant difference in depressive symptoms between CEs and non-CEs in Wave 1, but CEs experienced significantly greater depressive symptom scores in Wave 2. This indicates that caring is often a long-term task, with the associated responsibilities being both physically and emotionally draining, highly stressful, and time-consuming (Sherman, 2018). Consistent with the current literature (Glavin & Peters, 2015; Isenhour et al., 2012), our results show that CEs were more likely to report higher depressive symptoms compared with non-CEs in both waves, after controlling for possible confounders. CEs’ depressive symptom status may have been significantly influenced by the multiple roles of caring and employment. For example, CEs may experience increased pressure and stress due to the overwhelming demands of these roles. In the workplace, many CEs choose not to self-identify, likely due to a fear of being viewed as less committed and possibly being passed over for promotions or positions involving extensive travel (Wang et al., 2018; Sherman, 2018). Furthermore, CEs may be hesitant to seek assistance, which may worsen their negative health consequences, as they may feel isolated or lonely—feelings that are associated with depression, anxiety, substance abuse, and higher incidence of heart disease and stroke (Sherman, 2018).

Findings from the Wave 2 data suggest that social participation is negatively associated with CEs’ depressive symptoms, which is consistent with existing literature for informal carers in general (Sibalija et al., 2020; Pinto, 2016; Vlachantoni et al., 2013). These findings indicate that the protective moderating effect of social participation gradually becomes significant against depressive symptoms. Social participation, whether in leisure, recreational or spiritual activities or a favourite hobby, provides CEs a chance to de-stress and recharge. Linking this finding back to Pearlin et al.’s (1990) model, social participation can be a source of social support (Sibalija et al., 2020) and may also operate as a coping strategy (Hughes and Keller, 1992), recognizing that both coping and social support are modifiable factors in the model. There is also the possibility that CEs who have greater social participation are more likely to have an active social network and have greater opportunities to receive social support for caring, such as assistance from family members or friends, spiritual support from their faith community, or the benefits of strong interpersonal relationships (Kelley et al., 2017).

The findings from this study have important policy implications. CEs inevitably experience role conflict, affecting their time and energy. One novel social participation strategy available for many who continue to be employed is the adoption of carer-friendly workplace programs (CFWPs). These include a range of workplace initiatives, such as carer support groups, education and training seminars addressing topics of interest to CEs, a CE peer-support program, and specialized training for supervisors and managers to better understand the needs of CEs, as well as interventions to support a carer-friendly workplace culture (Wang et al., 2018; Ireson et al., 2018). Our study provides the evidence needed to ensure that CEs remain socially engaged, via social participation. Vlachantoni et al. (2013) suggest that time, energy and finances are barriers to social participation among carers, which is consistent with the work of Pinto (2016) and Innes et al. (2016), who studied barriers to leisure participation for people with dementia and their carers. The provision of certain CFWPs would alleviate many of the noted barriers while providing an opportunity to have CEs engage in social participation activities. CFWPs are unique to each workplace, given the sector-, size- and labour-specific variabilities, as well as workplace expectations (Sethi et al., 2017). Several easily accessible and time-limited complimentary tools are available to assist employers in creating CFWPs. The Canadian Standards Association Carer-Inclusive and Accommodating Standard (B-701), n.d. (available at https://www.csagroup.org/article/b701-17/), Handbook n.d. (available at https://www.csagroup.org/article/b701hb-18) and Quick-Study Guide, n.d. (available at https://ghw.mcmaster.ca/app/uploads/2020/07/Quick-Start-Implementation-Guide-_-Carer-Friendly-Workplace-Standard-Final.pdf) provide ready tools for employers to embark on tailoring a CFWP for their unique workplace (more tools can be found at https://ghw.mcmaster.ca/).

There are several significant limitations to this study. First, there are currently only two waves of CLSA data available, making it challenging to interpret a causal relationship between CEs’ social participation and depressive symptoms. Second, this study identifies CEs for older adults based on the relationship with the care recipients; this is due to the lack of information available in the CLSA specific to care recipients’ age. This may have led to a degree of bias in the analysis, even though the sensitivity analysis excluded the relationships that were unidentifiable and illustrated the robustness of our findings. Third, CLSA participants were not recruited based on their caring status and were not asked questions about the carer-related depressive symptoms. Therefore, the depressive symptoms of carers may be potentially overestimated or underestimated, given that the responses may reflect the overall mental health of CLSA participants. Fourth, CLSA lacks information about the health status of the care recipients. As we know, care recipient’s health status is an important confounder of carer depressive symptoms. Comparatively poorer care recipient health (i.e., dementia) causes higher carer burden and is thereby associated with a higher risk of developing depressive symptoms (Wang et al., 2011). However, we were not able to explore how the health status of the care recipient affects CE’s depressive symptoms.

## Conclusion

This research represents the first study using cross-sectional data that examines the association between caring, social participation, and depressive symptoms among Canadian carer-employees. We found that, over time, social participation is a protective moderator for CEs that reduces the negative outcomes of the many stressors experienced. The study highlights the importance of social participation in reducing CEs’ depressive symptoms, suggesting that encouraging and enhancing social participation are important. Undoubtedly, the workplace can play a role through the provision of carer-friendly workplace programs, which can be tailored to a variety of different organizations. Further research is needed to investigate what types of social participation have significant influences on CEs’ depressive symptoms, so as to provide more specific information for consideration by employers and policy makers alike.

### Contributions to knowledge

What does this study add to existing knowledge?

• The CLSA Wave 1 (2010) and Wave 2 (2014) data were used to carry out two cross-sectional analyses to examine the association between CEs and depressive symptoms, and to better understand the moderation effect of social participation.
• CEs were more likely to report higher depressive symptoms compared with non-CEs in both waves, after controlling for socio-economic demographic variables, general health status, and access to health care.
• Social participation is negatively associated with CEs’ depressive symptoms in Wave 2 but not in Wave 1, indicating that the protective moderating effect of social participation gradually becomes significant against depressive symptoms.

What are the key implications for public health interventions, practice or policy?

• The study provides the evidence needed to ensure that CEs remain socially engaged to combat depression.
• One novel social participation strategy is the adoption of carer-friendly workplace programs (CFWPs).
• CFWPs include a range of workplace initiatives, such as carer support groups, education and training seminars, peer-support programs and specialized training for supervisors/managers, as well as interventions to support a carer-friendly workplace culture.
• The complimentary CSA Carer-Inclusive and Accommodating Organizations Standard (https://www.csagroup.org/article/b701-17/) and Guide (https://www.csagroup.org/article/b701hb-18) provide a ready set of guidelines for workplaces to create a CFWP (available in French). For more tools, see https://ghw.mcmaster.ca/tools-and-curriculum/ghw.mcmaster.ca.

## References

[CR1] Arai Y, Kumamoto K, Mizuno Y, Washio M (2014). Depression among family caregivers of community-dwelling older people who used services under the Long Term Care Insurance program: A large-scale population-based study in Japan. Aging & Mental Health.

[CR2] Caldwell J (2008). Health and access to health care of female family caregivers of adults with developmental disabilities. Journal of Disability Policy Studies..

[CR3] Canadian Longitudinal Study on Aging. (2011). Sampling and computation of response rates and sample weights for the tracking (telephone interview) participants and comprehensive participants.

[CR4] Carer-Inclusive Standard for Workplaces: B701-F17 – Carer-inclusive and accommodating organizations. (n.d.). https://www.csagroup.org/article/b701-17/.

[CR5] Day MD (2017). Gender, spousal caregiving, and depression: Does paid work matter?. Journal of Marriage and Family.

[CR6] de Oliveira, G. R., Neto, J. F., de Camargo, S. M., Lucchetti, A. L. G., Espinha, D. C. M., & Lucchetti, G. (2015). Caregiving across the lifespan: comparing caregiver burden, mental health, and quality of life. *Psychogeriatrics, 15*(2), 123–132. 10.1111/psyg.12087 25521215

[CR7] Duxbury L, Higgins C, Smart R (2011). Elder care and the impact of caregiver strain on the health of employed caregivers. Work.

[CR8] Glavin P, Peters A (2015). The costs of caring: Caregiver strain and work-family conflict among Canadian workers. Journal of Family and Economic Issues.

[CR9] Health Quality Ontario. (2017). Measuring up 2017: A yearly report on how Ontario’s health system is performing. Toronto: Queen’s Printer for Ontario.

[CR10] Hughes S, Keller MJ (1992). Leisure education. Journal of Gerontological Social Work.

[CR11] Implementation Guide: B701HB-18 – Helping worker-carers in your organization. (n.d.) https://www.csagroup.org/article/b701hb-18.

[CR12] Innes A, Page SJ, Cutler C (2016). Barriers to leisure participation for people with dementia and their carers: An exploratory analysis of carer and people with dementia’s experiences. Dementia.

[CR13] Ireson R, Sethi B, Williams A (2018). Availability of caregiver-friendly workplace policies (CFWPs): An international scoping review. Health & Social Care in the Community.

[CR14] Isenhour LC, Stone DL, Lien D, Zhang M, Griffeth RW, Fried DD (2012). Work-family conflict and individual consequences. Journal of Managerial Psychology.

[CR15] Kelley DE, Lewis MA, Southwell BG (2017). Perceived support from a caregiver’s social ties predicts subsequent care-recipient health. Preventive Medicine Reports.

[CR16] Kim Y, Carver CS, Rocha-Lima C, Shaffer KM (2011). Depressive symptoms among caregivers of colorectal cancer patients during the first year since diagnosis: A longitudinal investigation. Psycho-Oncology.

[CR17] Luchsinger JA, Tipiani D, Torres-Patiño G, Silver S, Eimicke JP, Ramirez M, Teresi J, Mittelman M (2015). Characteristics and mental health of Hispanic dementia caregivers in New York City. American Journal of Alzheimer's Disease and Other Dementias.

[CR18] O’Reilly D, Connolly S, Rosato M, Patterson C (2008). Is caring associated with an increased risk of mortality? A longitudinal study. Social Science & Medicine.

[CR19] Pearlin LI, Mullan JT, Semple SJ, Skaff MM (1990). Caregiving and the stress process: An overview of concepts and their measures. The Gerontologist.

[CR20] Piercy KW, Fauth EB, Norton MC, Pfister R, Corcoran CD, Rabins PV, Lyketsos C, Tschanz JT (2013). Predictors of dementia caregiver depressive symptoms in a population: The Cache County Dementia Progression Study. The Journals of Gerontology: Series B.

[CR21] Pinto JM (2016). Barriers to social participation in caregivers of older people: A systematic review. Research in Health Science.

[CR22] Quick-Study Guide. (n.d.). https://ghw.mcmaster.ca/app/uploads/2020/07/Quick-Start-Implementation-Guide-_-Carer-Friendly-Workplace-Standard-Final.pdf.

[CR23] Radloff, L. S. (1977). The CES-D scale: A self-report depression scale for research in the general population. *Applied Psychological Measurement, 1*(3), 385–401.

[CR24] Robison J, Fortinsky R, Kleppinger A, Shugrue N, Porter M (2009). A broader view of family caregiving: Effects of caregiving and caregiver conditions on depressive symptoms, health, work, and social isolation. The Journals of Gerontology. Series B, Psychological Sciences and Social Sciences.

[CR25] Rubin, D. B. (2004). *Multiple imputation for nonresponse in surveys* (Vol. 81). John Wiley & Sons.

[CR26] Sethi B, Williams A, Ireson R (2017). Supporting caregiver employees: managers’ perspective in Canada. International Journal of Workplace Health Management.

[CR27] Sherman C (2018). Rising to the challenge: What employers can do to support caregiver employees. Benefits Quarterly.

[CR28] Sibalija J, Savundranayagam MY, Orange JB, Kloseck M (2020). Social support, social participation, & depression among caregivers and non-caregivers in Canada: A population health perspective. Aging & Mental Health.

[CR29] Sinha, M. (2013). Portrait of caregivers, 2012. Statistics Canada Catalogue no. 89-652-X-No.001. Ottawa. Industry Canada, 21 pp. www.statcan.gc.ca/pub/89-652-x/89-652-x2013001-eng.pdf.

[CR30] Vlachantoni A, Evandrou M, Falkingham J, Robards J (2013). Informal care, health and mortality. Maturitas.

[CR31] Wang L, Williams A, Kitchen P (2018). Health of caregiver-employees in Canada. International Journal of Workplace Health Management.

[CR32] Wang YN, Shyu Y-UL, Chen M-C, Yang P-S (2011). Reconciling work and family caregiving among adult-child family caregivers of older people with dementia: Effects on role strain and depressive symptoms. Journal of Advanced Nursing.

[CR33] Williams AM, Tompa E, Lero DS, Fast J, Yazdani A, Zeytinoglu IU (2017). Evaluation of caregiver-friendly workplace policy (CFWPs) interventions on the health of full-time caregiver employees (CEs): Implementation and cost-benefit analysis. BMC Public Health.

